# Electrochemical Fluorination and Radiofluorination of Methyl(phenylthio)acetate Using Tetrabutylammonium Fluoride (TBAF)

**DOI:** 10.1149/2.0941709jes

**Published:** 2017-07-14

**Authors:** Mehrdad Balandeh, Christopher Waldmann, Daniela Shirazi, Adrian Gomez, Alejandra Rios, Nathanael Allison, Asad Khan, Saman Sadeghi

**Affiliations:** a Department of Molecular & Medical Pharmacology, University of California, Los Angeles, California 90095, USA; b Department of Chemistry and Biochemistry, UCLA, Los Angeles, California 90095-1569, USA

## Abstract

Electrochemical fluorination of methyl(phenylthio)acetate was achieved using tetrabutylammonium fluoride (TBAF). Electrochemical fluorination was performed under potentiostatic anodic oxidation using an undivided cell in acetonitrile containing TBAF and triflic acid. The influence of several parameters including: oxidation potential, time, temperature, sonication, TBAF concentration and triflic acid concentration on fluorination efficiency were studied. It was found that the triflic acid to TBAF concentration ratio plays a key role in the fluorination efficiency. Electrochemical fluorination resulted in formation of mono-fluorinated methyl 2-fluoro-2-(phenylthio)acetate verified by gas chromatography–mass spectrometry (GC-MS) and nuclear magnetic resonance (NMR) Spectroscopy. Under optimum conditions 44 ± 3% mono fluorination yield was obtained after a 30 min electrolysis. Electrochemical radiofluorination for the synthesis of methyl 2-[^18^F]fluoro-2-(phenothio) acetate was also achieved with the same optimized electrochemical cell parameters where TBAF was first passed through an anion exchange resin containing fluorine-18. A radiochemical fluorination efficiency of 7 ± 1% was achieved after 30 min of electrolysis.

Incorporation of fluorine into a lead molecule can have a positive impact on metabolic stability, pKa, intrinsic potency, membrane permeability and pharmacokinetic of bioactive molecules.^1–6^ Organofluorine molecules can rarely be found in nature and hence the introduction of a fluorine atom into a naturally occurring organic molecule requires the development of appropriate synthetic methods developed in the lab. Fluorine gas and fluorinating agents derived from it have widely been used as the source of fluorine atom for fluorination of organic compounds.^7–10^ However, fluorination of organic substances using fluorine gas is difficult because fluorine gas is highly toxic and reactive. Furthermore, the ^18^F isotope of fluorine, which has been established as the most promising isotope for Positron Emission Tomography (PET), is most accessible and practical for PET tracer development in 18F-fluoride form produced via a ^18^O-H_2_O(p,n)^18^F nuclear reaction in a cyclotron.^11^ PET has extensive clinical applications in early disease diagnosis, treatment progression monitoring as well as in drug discovery and development.^12^ Despite the synthesis of a wide variety of ^18^F labeled PET probes, their clinical translation is often hindered due to a lack of viable late-stage synthesis methods with ^18^F-fluoride and the 110-minute half-life of the isotope.^13^ The biggest roadblock in making a wider scope of fluorinated molecules easily accessible, is that precursors with no positive charge at the site of fluorine labeling are not readily amenable to nucleophilic substitution reactions with fluoride.^14^ Development of PET tracers and availability of fluorinated bioactive molecules synthesized by nucleophilic fluoride would benefit from a convenient late stage fluorination method.^15^ Electrochemical nucleophilic fluorination of organic molecules has been reported as a powerful method for introduction of the fluorine atom into organic compounds.^16–18^ Electrochemical fluorination has commonly been performed in solutions containing an excess of poly HF salts such as Et_3_N.3HF and Et_3_N.4HF as a fluoride source.^19–22^ However, HF salts are expensive, toxic and corrosive. Tetrabutylammonium fluoride (TBAF) is a source of fluoride which is less toxic, easier to handle and also inexpensive compare to HF salts, making it a suitable alternative for electrochemical fluorination. A further advantage of using TBAF, instead of HF salts as a source of fluoride, is the traditional use of ^18^F-TBAF in radiofluorination.^23^ Previous attempts at electrochemical radiofluorination with ^18^F-poly-HF salts, which severely limits specific activity and places a theoretical limit on radiochemical yield, have been reported.^24,25^ However, previous reports on the use TBAF for electrochemical fluorination of phenyl(2,2,2-trifluoroethyl)sulfane, have not been successful.^16^ [^18^F]fluoride in form of ^18^F-TBAF in this report was obtained by first trapping [^18^F]fluoride anion on an anion exchange resin in order to remove the water, and subsequent elution of [^18^F]fluoride from the cartridge using tetrabutylammonium fluoride.

Electrochemical fluorination using TBAF was only made possible with the addition of trifluoromethanesulfonic (triflic) acid during electrolysis. Triflic acid is a known super acid whose conjugate base is a very weak nucleophile.^26^ The addition of triflic acid may form HF molecules that can participate in electrochemical fluorination, while the very weak conjugate base of triflic acid will not react with the intermediate carbocations formed during electrochemical oxidation.

In this paper, we present the successful electrochemical fluorination of methyl(phenylthio)acetate using TBAF as a source of fluorine. Furthermore, ^18^F-methyl 2-fluoro-2-(phenylthio)acetate was radiosynthesized using ^18^F-TBAF as a source of fluorine. The products were detected and analyzed using HPLC, GC-MS and NMR (see supporting information). Effect of several parameters such as electrolysis potential, time, temperature, triflic acid concentration and TBAF concentration were investigated and optimized.

## Experimental

High-resolution mass spectra and chromatograms were obtained with an Agilent 5975C TAD inert MSD mass spectrometer coupled with an Agilent 7890A gas chromatograph. Cyclic voltammeteric (CV) and electrosynthesis experiments were performed using the Metrohm PGSTAT128N electrochemical workstation. All CVs were performed at room temperature using a 200 mV/s scan rate.

Radiofluorination conversion was measured using Radio-thin-layer-chromatography (radio-TLC). Radio-TLC was performed on silica plates (TLC Silica gel 60 W F_254_s, Merck). After dropping a sample volume (∼1–5 μL) using a glass capillary, the plate was developed in the mobile phase (ACN). Chromatograms were obtained using a radio-TLC scanner (miniGita Star, Raytest).

Analytical High Performance Liquid Chromatography (HPLC), equipped with a UV and gamma detector was used to determine radiochemical purity (RCP) of the radio-fluorinated product. HPLC was performed using a 1200 Series HPLC system (Agilent Technologies) equipped with a GabiStar flow-through gamma detector (Raytest). Data acquisition and processing was performed using GINA Star Software version 5.9 Service Pack 17 (Raytest). Typically, 20 μL of radioactive sample was diluted with 180 μL of ACN and 5–20 μL of this solution was injected for HPLC analysis. Column: Phenomenex Luna 5u C18 (2) 100 A, 250 × 4.6 mm, 5 micron. Gradient: A = ACN; B = water; flow rate = 1.8 mL/min; 0–12 min 90% B to 5% B, 12–13 min 5% B, 13–14 min 5% B to 90% B.

Radio-TLC chromatograms were used to measure radiochemical conversions (RCC). RCP and RCC were measured by dividing the area under the curve (AUC) for the desired product by the sum of AUC for all peaks. The TLC purity accounts for unreacted ^18^F-fluoride while the HPLC purity corrects for radiochemical side-products. The radiochemical fluorination efficiency (RCFE) was determined by the equation: RCFE = TLC RCC × HPLC RCP.

The GC-MS, TLC and HPLC analysis were performed on crude samples, and the reported yields in optimization studies are based on the quantification of GCMS results. The reaction products were HPLC purified and isolated for purposes of proton and fluorine NMR analysis for further identification (see supporting information).

The electrochemical fluorination and CVs were carried out using a conventional undivided 3-electrode cell with two platinum wires (length = 200 mm, diameter = 0.33 mm) as working and counter electrode and Ag/Ag^+^ reference electrode in a 13.2 ml solution containing dry ACN (11 mL), 2 ml of 1 M TBAF solution in tetrahydrofuran (THF) (154 mM TBAF final concentration), 120 μl of triflic acid (104.6 mM final concentration) and 50.8 μl of methyl(phenylthio)acetate (25 mM final concentration). The reference electrode solution was 10 mM AgNO_3_ plus 100 mM tetrabutylammonium perchlorate in dry ACN. The reference electrode solution was separated from the reaction mixture by porous glass frit.

The counter electrode and working electrode were cleaned before each experiment using potential cycling in 1 M sulfuric acid solution in water. The electrodes were cycled between −2 V and 2 V (2 electrode configuration) 10 times before each experiment.

Trifluoromethanesulfonic acid (triflic acid, CF_3_SO_3_H, 99%) and methyl(phenylthio)acetate (C_9_H_10_O_2_S, 99%) were purchased from Oakwood Chemical. Acetonitrile (ACN, anhydrous, 98%), tetrabutylammonium fluoride solution 1.0 M in THF (TBAF solution, ~5 wt% water) and platinum wire (99.9%) were purchased from Sigma-Aldrich.

Electrolysis was performed using a constant potential technique while the solution was stirred at 300 rpm. In order to prevent formation of polymerized products on the working electrode, the polarity of the electrode was alternated every 60 s between the chosen fluorination potential and −0.6 V; electrode was kept at −0.6 V for 5 s.

No-carrier-added ^18^F-fluoride was produced by the (p,n) reaction of ^18^O–H_2_O (84% isotopic purity, Medical Isotopes) in a RDS-112 cyclotron (Siemens) at 11 MeV using a 1 mL tantalum target with havar foil.^27^ The radioactive isotope was trapped on an anion exchange resin by passing through the 1ml of bombarded ^18^O–H_2_O. Most of the water on the resin was removed by washing with 10 mL of anhydrous ACN and drying with ultra-pure N_2_ for 10 min. [^18^F]fluoride was subsequently eluted out from the cartridge with a 2 ml solution containing 0.5 mmol TBAF in THF + ACN solution (1:1). In a typical experiment, approximately 5 mCi was eluted from the anion exchange cartridge in ^18^F-TBAF form in dry ACN.

## Results and Discussion

**Figure 1:**
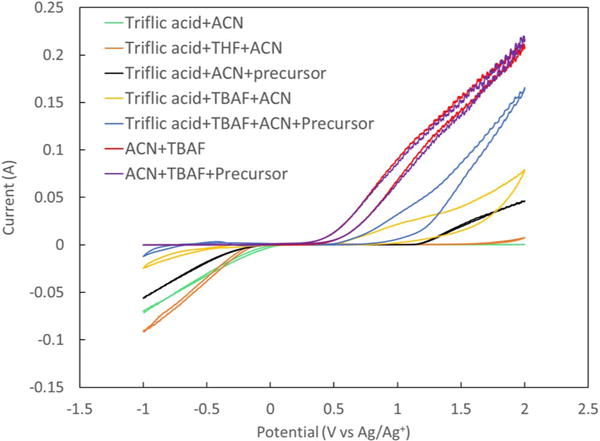
CVs of different combination of materials were used in the electrochemical fluorination of methyl(phenylthio)acetate. The CVs were run using 200 mv.s^−1^ at room temperature (21°C).

Figure 1 shows CVs of different combination of chemicals used in the electrochemical fluorination experiments. It can be seen that the CV of ACN + precursor shows a very small cathodic or anodic current between −1 V to 2 V. While CVs of ACN + triflic acid and ACN + triflic acid + THF also shows very small anodic current at potentials higher than 0 V vs Ag/Ag^+^, a high cathodic current can be observed at potentials below 0 V vs Ag/Ag^+^, which can be attributed to the hydrogen evolution on the working electrode. Although ACN + precursor and ACN + triflic acid don’t show any anodic current at positive potentials, a combination of these (ACN + triflic acid + precursor) displays an anodic current at potentials higher than 1 V vs Ag/Ag^+^. This can be due to the oxidation of the precursor, promoted by the addition of triflic acid. Furthermore, CVs of ACN + TBAF and ACN + TBAF + precursor are very similar, in the way that they don’t show any cathodic current, while at potentials higher than 0.5 V vs Ag/Ag^+^ an increase in anodic current can be observed. Similarity in anodic current is due to the high oxidation current from the TBAF solution which has a lower onset potential and occurs at much higher rate compared to the oxidation of the precursor and masks any additional negligible current contribution from the oxidation of the precursor. The addition of triflic acid to these solutions causes a sharp decrease in the anodic current, which is in line with the proposed reaction of triflic acid with TBAF, with hydrogen fluoride as a possible product of this reaction.

**Figure 2:**
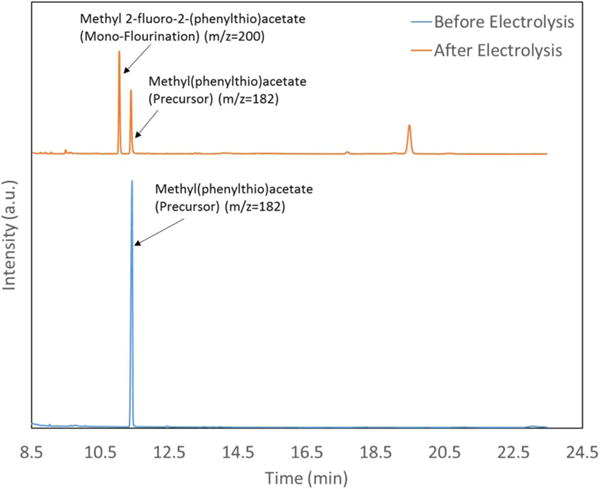
GC-MS chromatogram of the solution before and after electrochemical fluorination. The solution contains 25 mM of **1**, 154 mM TBAF and 104.6 mM of triflic acid in acetonitrile.

**Figure 3:**
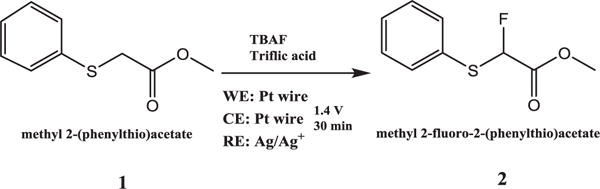
Schematic of the electrochemical fluorination of methyl-2(phenylthio)acetate **1** using TBAF.

The anodic fluorination of methyl(phenylthio)acetate was carried out at constant potential in an undivided cell. The products were analyzed using GC-MS. Figure 2 shows a representative GC-MS chromatogram of the solution before and after electrolysis at 1.4 V for 30 min at room temperature. The chromatogram of the solution before electrolysis shows only one peak for the precursor with the m/z equal to 182. Figure 2 shows that after electrolysis the precursor peak area has decreased and the product peak is observed at 11 min with the m/z of 200. The full mass spectra for these peaks are presented in the supporting information. The 11 min product is attributed to methyl 2-fluoro-2-(phenylthio)acetate (**2**) (monofluorination). The schematic for the electrochemical fluorination of methyl(phenylthio)acetate (**1**) has been shown in the Figure 3.

A linear calibration curve for the precursor was obtained and used in the quantification of precursor consumption and production yield of the product (supporting information). The purified sample of the product was further identified by proton and fluorine NMR and provided matching retention time on the GCMS. (see supporting information).

**Figure 4:**
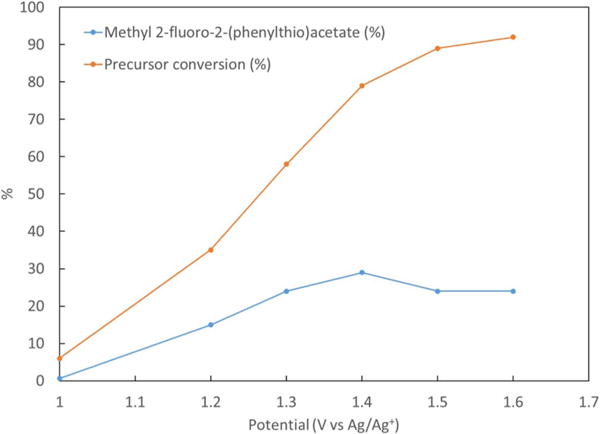
Effect of electrolysis potential on the yield of product and precursor conversion. Synthesis has been performed at the constant time of 30 min, using ACN solution containing 154 mM of TBAF, 25 mM of precursor **1** and 104.6 of triflic acid.

**Figure 5:**
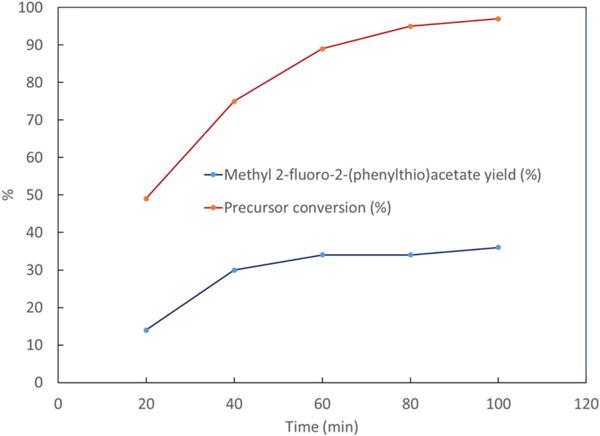
Effect of time on the yield of product and precursor conversion. Synthesis has been performed at constant potential of 1.4 V vs Ag/Ag^+^, using ACN solution containing 154 mM of TBAF, 25.1 mM of **1** and 104.56 of triflic acid.

Product yields and precursor conversion of the electrofluorinated samples are presented in Figure 4 with different oxidation potentials and in Figure 5 with different electrolysis times. Experiments were performed in triplicates. It can be seen that by increasing the potential from 1 V to 1.4 V vs Ag/Ag^+^ both the yields and precursor conversion increase. However, further increase in the potential reduces yields. This may be due to enhanced product oxidation and decomposition at potentials higher than 1.4 V vs Ag/Ag^+^. It can also be seen from Table II that by increasing electrolysis time, the yield increases with time until a saturation is reached at 36% yield for **2** at 100 min when most of the precursor has been consumed. A shorter electrolysis time of 30 min with a comparable yield of 29% was selected for further optimization. This was selected in preparation for radiochemical fluorination with ^18^F-fluoride, which has a 110 min half-life, and benefits from increased non-decay-corrected radiochemical yield with shorter synthesis times.

**Figure 6:**
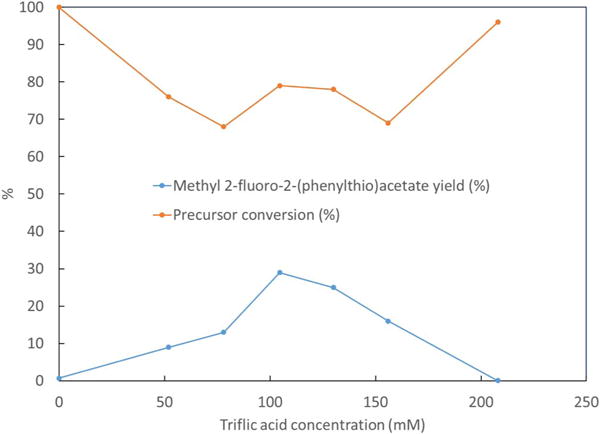
Effect of triflic acid concentration on the product yield and precursor conversion. Synthesis has been performed at constant time and potential of 30 min and 1.4 V vs Ag/Ag^+^, using ACN solution containing triflic acid, 154 mM of TBAF and 25 mM of **1**.

The effect of triflic acid concentration was also examined. The results are shown in Figure 6. When acid concentration increases from 0 to 104.6 mM yield of **2** increases from 0.7% to 29%. Further increase in acid concentration beyond 104.6 mM results in a decrease in yield of **2** until a yield of 0.03% is reached using 208 mM triflic acid.

Electrofluorination was performed using triflic acid, acetic acid and sulfuric acid to study the effect of acid type on product yield. The results are shown in the Table I, with triflic acid providing the highest precursor conversion and yield for **2**.

Table II shows the results of the electrofluorination experiments performed at three different temperatures. It was observed that elevating the temperature has a positive effect on the electrofluorination yield. For instant, the yield of the **2** could be increased from 8% to 44% by increasing temperature from 0 °C to 60°C. Elevating the temperature can enhance the diffusion of the molecules in the solution leading to increased yields. The solution was also sonicated in order to confirm if promoting convection in the solution could enhance the yield. Sonication at room temperature increased the yield of **2** from 29% to 42%, a similar gain in yield as was observed with the increase in temperature.

It was also observed that triflic acid to TBAF concentration ratio has a crucial effect on the electrofluorination yield. As the TBAF concentration was decreased and triflic acid concentration was maintained constant, much lower yield of **2** was observed as compared to when TBAF and triflic acid concentrations were proportionally decreased together to maintain a constant ratio. The triflic acid to TBAF ratio, with 154 mM of TBAF, was optimized at 0.68 from the data in Figure 6.

To study the effect of acid to TBAF concentration ratio, two sets of experiments were performed. In the first set the concentration of TBAF was changed and the triflic acid concentration was kept constant, in the second set the TBAF concentration was changed and the triflic acid concentration also was changed in order to keep the triflic acid to TBAF concentration ratio constant at 0.68. The results are compared and presented in the Table III for TBAF concentration ranging from 154 mM to 10 mM.

One of the advantages of using TBAF as a fluoride source in electrochemical fluorination is its compatibility and traditional use in radiofluorination with ^18^F-fluoride. Compared with poly-HF salts, TBAF introduces fewer carrier ^19^F-fluoride molecules, increasing specific activity and radiochemical yield, which is measured with respect to ^18^F-fluoride incorporation into the desired product. Since the concentration of ^18^F-fluoride used in radiochemistry is negligibly small, we studied the effect of decreasing TBAF concentration on electrofluorination yield. The results are shown in Table IV, indicating that lowering the fluoride concentration from 308 mM to 154 mM does not significantly change the yield of **2**, however a further decrease in fluoride concentration below 154 mM decreases yield of **2**. While lowering fluorine concentration below the concentration of precursor would limit the theoretical chemical yield, the decrease in yield at higher concentrations may be attributed to the limited lifetime of carbocations formed at the surface of the working electrode. At lower concentrations of TBAF, cationic intermediates created on the anode with no fluoride in close vicinity, will have a diminishing chance to react with the fluoride nucleophile before they undergo side reactions. Table IV also shows the yield of **2** based on the fluoride concentration. It can be seen that by lowering the fluoride concentration from 308 mM to 10 mM the yield of **2** increases, while a further decrease in concentration of fluoride below 10 mM causes a drastic decrease in the yield of **2**.

Electrochemical radiofluorination of the **1** was successfully achieved using the optimized parameters obtained from the cold electrofluorination experiments. (1.4 V, 30 min, 60°C, 154 mM of TBAF, 25 mM of **1** and 104.6 mM of triflic acid). Radiochemical fluorination efficiency obtained by TLC and gamma HPLC was 7 ± 1% (n = 3). The chemical yield (based on the initial precursor concentration) obtained from the decayed samples analyzed by GC-MS showed a yield of 43% ± 3% (n = 3), which would predict a fluorination yield (based on the initial fluorine concentration) of 7 ± 1% (n = 3) for the mono-fluorinated product, in line with the radiochemical yields obtained.

## Conclusions

For the first time, electrochemical fluorination of methyl(phenylthio)acetate has been achieved using TBAF as a source of fluorine under controlled potentiostatic conditions. It was observed that the use of triflic acid along with TBAF is crucial for successful fluorination and that the TBAF to triflic acid concentration ratio plays a key role in the process. Electrochemical cell parameters such as potential, electrolysis time, and temperature as well concentrations of fluoride source and triflic acid were optimized. CVs guided the selection of oxidation potentials and our understanding of the electrochemical oxidation/reduction response of the system. The optimum oxidation potential of **1** was found to be 1.4 V vs Ag/Ag^+^. Potentials higher than 1.4 V vs Ag/Ag^+^ resulted in lower yields, likely due to the breakdown of the product. Fluorination at potentials between 1.3 V vs Ag/Ag^+^ and 1.1 V vs Ag/Ag^+^ required a long time to achieve acceptable yields, which isn’t desirable for radioelectrochemical fluorination with ^18^F-fluoride, which has a 110 min half-life. It was also observed that elevating temperature and sonication could enhance the yield. The highest yield for the mono fluorinated product at 44% was obtained after 30 min of electrolysis at 1.4 V vs ag/Ag^+^ using an ACN solution containing 154 mM of TBAF, 25 mM of precursor **1** and 104.6 mM of triflic acid at 60°C.

Electrochemical radiofluorination of methyl 2- [^18^F]fluoro-2-(phenothio) acetate was confirmed by GC-MS, radio-TLC and HPLC analysis. A radiochemical fluorination efficiency of 7 ± 1% was achieved under the same conditions as the optimized cold reaction for the mono fluorinated product.

## Supplementary Material

supporting information

## Figures and Tables

**Table I:** Effect of acid type on the product yield and precursor conversion. Synthesis was performed at constant time and potential of 30 min and 1.4 V vs Ag/Ag^+^, using ACN solution containing 154 mM of TBAF, 25 mM of 1 and 104.6 mM acid.

Type of Acid	Methyl 2-fluoro-2-(phenylthio) acetate yield (%)	Precursor conversion (%)
Trifluoromethanesulfonic Acid	29 ± 2	74 ± 14
Sulfuric Acid	3.0 ± 0.2	11 ± 2
Acetic Acid	0.3 ± 0.1	42 ± 8

**Table II:** Effect of temperature on the product yield and precursor conversion. Synthesis was performed at constant time and potential of 30 min and 1.4 V vs Ag/Ag^+^, using ACN solution containing 154 mM of TBAF, 25 mM of 1 and 104.6 of triflic acid.

Temperature (°C)	Methyl 2-fluoro-2-(phenylthio)acetate (%)	Precursor conversion (%)
0	7.6 ± 0.5	65 ± 12
21	29 ± 2	74 ± 14
60	44 ± 3	63 ± 12

**Table III:** Effect of triflic acid to TBAF concentration ratio on the product yield and precursor conversion. Synthesis was performed at constant time and potential of 30 min and 1.4 V vs Ag/Ag^+^, using ACN solution containing triflic acid, TBAF, and 25 mM of 1.

TBAF Concentration (mM)	Acid Concentration (mM)/TBAF concentration (mM)	Methyl 2-fluoro-2-(phenylthio)acetate yield (%)	Precursor conversion (%)
154	0.68	29 ± 2	74 ± 14
	1.36	0.03 ± 0.01	95 ± 3
100	0.68	21 ± 2	68 ± 12
	1.04	5.0 ± 0.3	69 ± 13
25	0.68	6.0 ± 0.4	37 ± 7
	4.16	0	94 ± 3
10	0.68	3.0 ± 0.2	24 ± 4
	10.40	0	74 ± 13

**Table IV:** Effect of TBAF concentration on the product yield and precursor conversion. Synthesis was performed at constant time and potential of 30 min and 1.4 V vs Ag/Ag^+^, using ACN solution containing 154 mM of TBAF, 25 mM of 1 and the ratio of triflic acid to TBAF concentration was kept constant at 0.68.

TBAF Concentration (mM)	Methyl 2-fluoro-2-(phenylthio)acetate yield (%)	Precursor conversion (%)	Methyl 2-fluoro-2-(phenylthio)acetate yield (%)^a^
308	29 ± 2	76 ± 14	2.5 ± 0.2
154	29 ± 2	74 ± 14	5.0 ± 0.3
100	21 ± 2	68 ± 12	5.0 ±0.3
25	6.0 ± 0.4	37 ± 7	5.5 ± 0.4
10	3.0 ±0.2	24 ± 4	7.5 ± 0.5
5	0.15 ± 0.01	10 ± 2	0.75 ± 0.05

a Yield was calculated using initial fluorine concentration (product concentration/initial fluorine concentration).
