# Supplementary material for: Electrochemical Fluorination and Radiofluorination of Methyl(phenylthio)acetate Using Tetrabutylammonium Fluoride (TBAF)
Source: J Electrochem Soc. Author manuscript; Available in PMC 2017 Sep 7. (PMC5589193; doi:10.1149/2.0941709jes)
Supplement: supporting information [file NIHMS901114-supplement-supporting_information.docx]

GC-MS Summary Method: Methyl-2-(phenylthio)-acetate

Mass Spectrum:

The mass spectrum was set to electron ionization mode with a voltage of 1.9 kV. The Mass range was 50-300 (amu).

Inlet:

Inlet was set at 200°C and had 1:10 split ratio

Oven:

Oven was set to 50°C and held for 1 min, then increased to 200°C at a rate of 20°C/min and held for 15 mins.

Columns:

Agilent 122-7033, 240°C; 30 m x 250 μm x 0.5 μm delivered a constant flow of 1 mL/min to the transfer column. The transfer column Agilent G3185-60062, 450°C; 0.17 m x 100 μm x 0 μm delivered a constant flow of 1.5 mL/min to the source.

All the samples were diluted by a factor of 3 for the GC-MS analysis.

The GC-MS method had an 8.5 min solvent delay in order to enhance the MS filament lifetime.

The identity of the product **2** was also determined by nuclear magnetic resonance spectroscopy (NMR). Product **2** was formulated in MeCN-d3 (Cambridge Isotope Laboratories) and NMR spectra obtained on a Bruker AV400 (400 MHz for 1H). ^1^H and ^19^F chemical shifts are reported in parts per million (ppm) using the solvent resonance as an internal reference.

Figure 1. is the GC calibration plot used in the quantification of precursor **1** conversion and formation of product **2**. Figure 2 shows the GCMS chromatogram and mass spectrum of the pure precursor 1. Figure 3 shows the GCMS chromatogram and mass spectrum of the product **2**, purified post electrochemical synthesis using a semi-preparative HPLC column. Figure 4 shows the gamma + UV HPLC chromatograms of the crude product post radio-electrochemical synthesis. Figure 5 shows the gamma TLC trace of the crude product post radio-electrochemical synthesis. While the TLC plate can be used to accurately quantify the percentage of the ^18^F-fluoride incorporated into an organic compound vs unreacted ^18^F-fluoride, it does not have the resolution to separate the 2 fluorinated organics observed in the gamma-HPLC trace in figure 4. Figure 6 shows the proton NMR of the purified product **2** for further identification of the fluorinated products obtained by the electrochemical fluorination method. Figure 7 is the fluoride NMR of the same sample in figure 7. ^1^H and ^19^F NMR spectroscopic data for product **2** were identical to that previously reported ^1^.

Figure 1. The GC calibration plot used in the quantification of precursor **1** conversion and formation of product **2**.

| 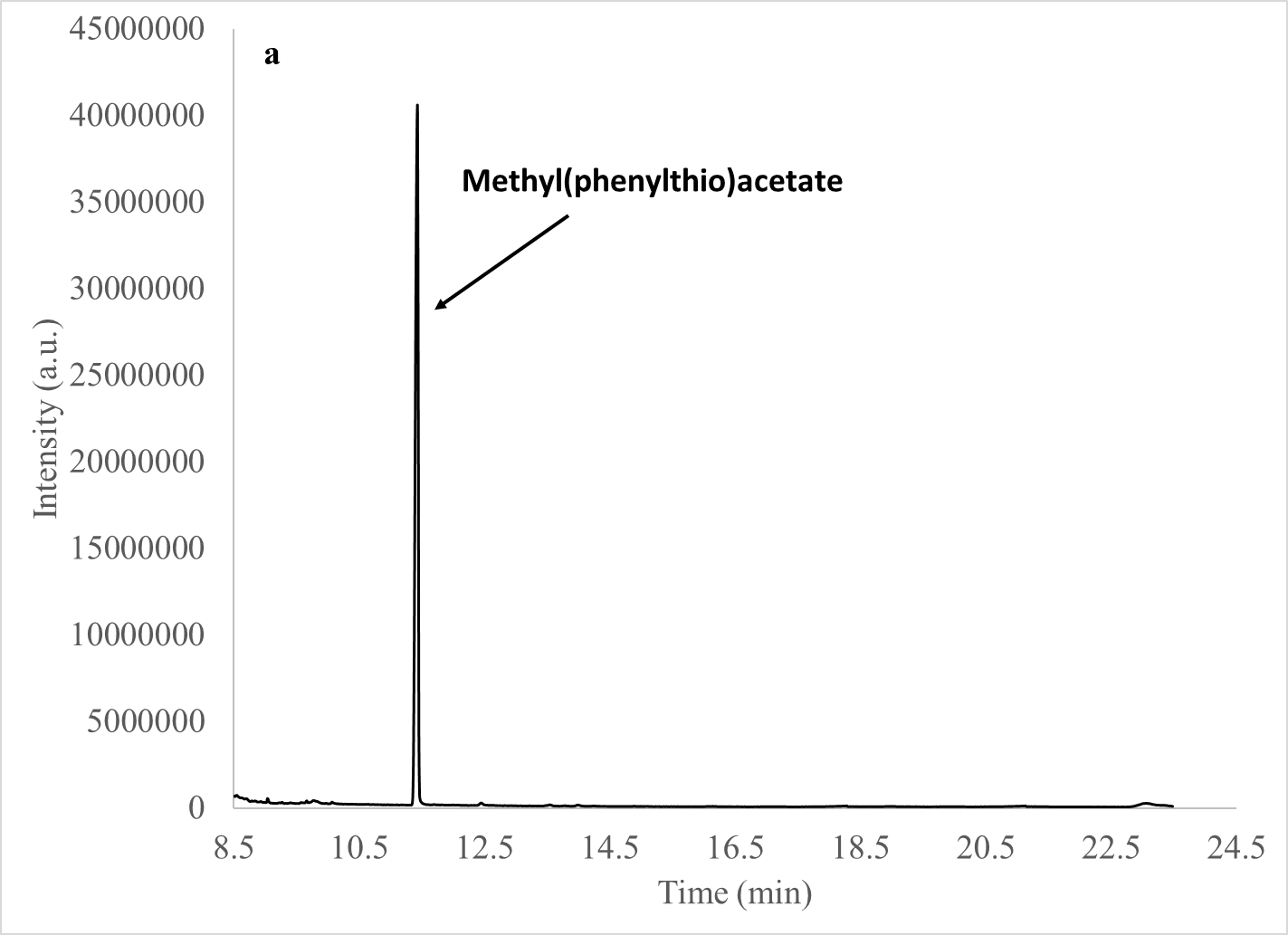 |
| --- |
| 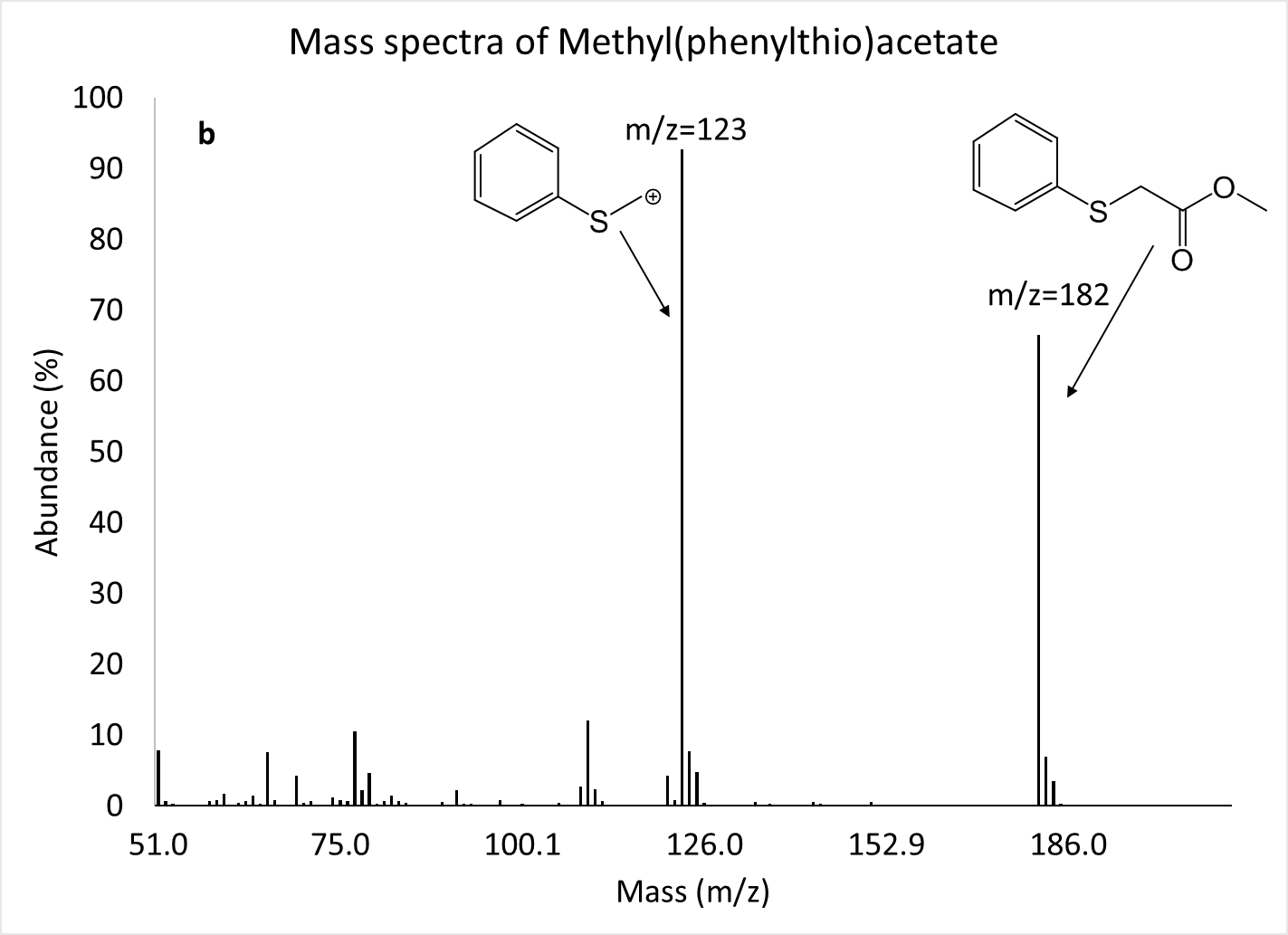 |

. Figure 2. The a) GC-MS chromatogram and b) mass sectrum of the pure precursor **1**.

| 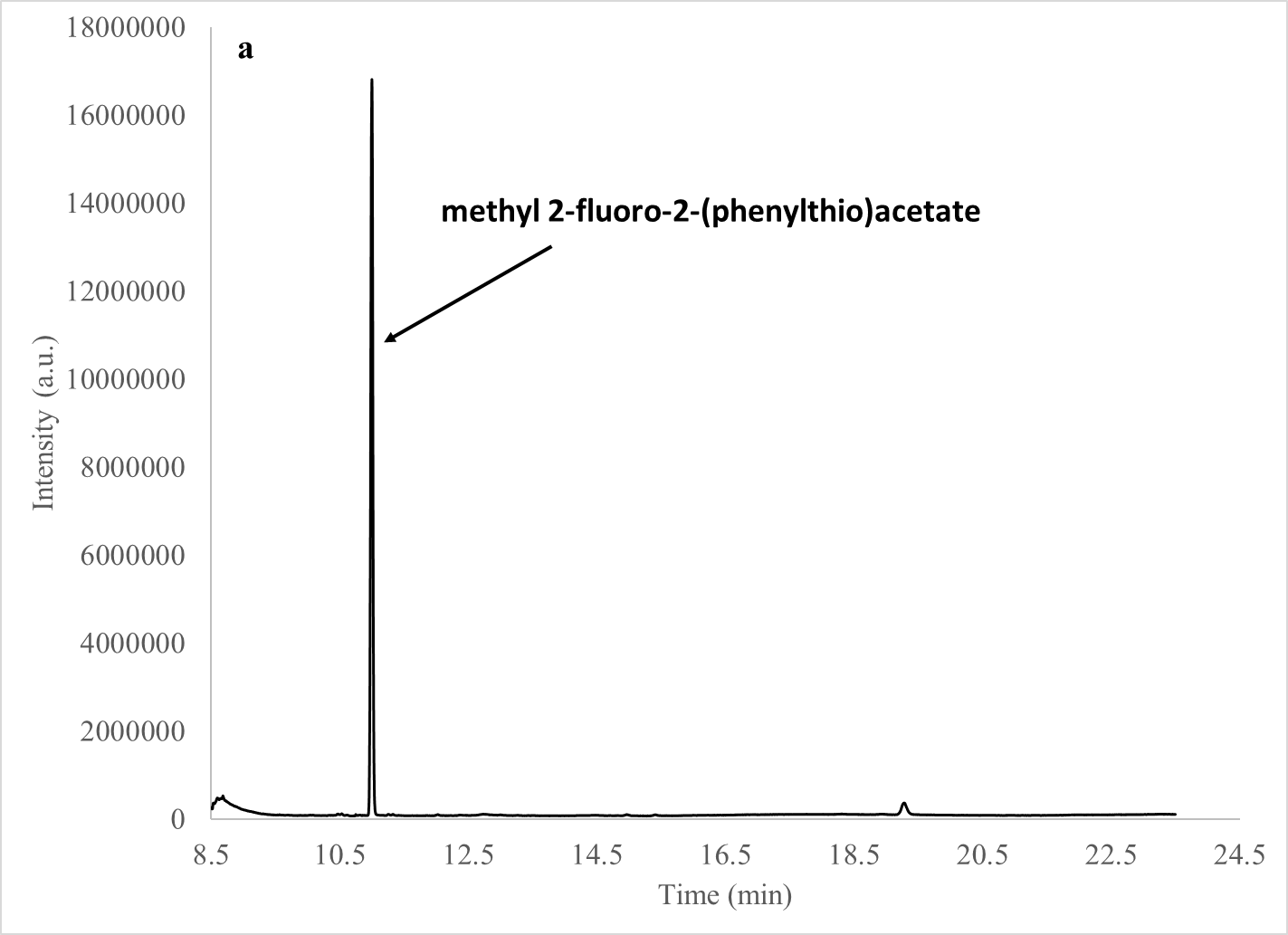 |
| --- |
| 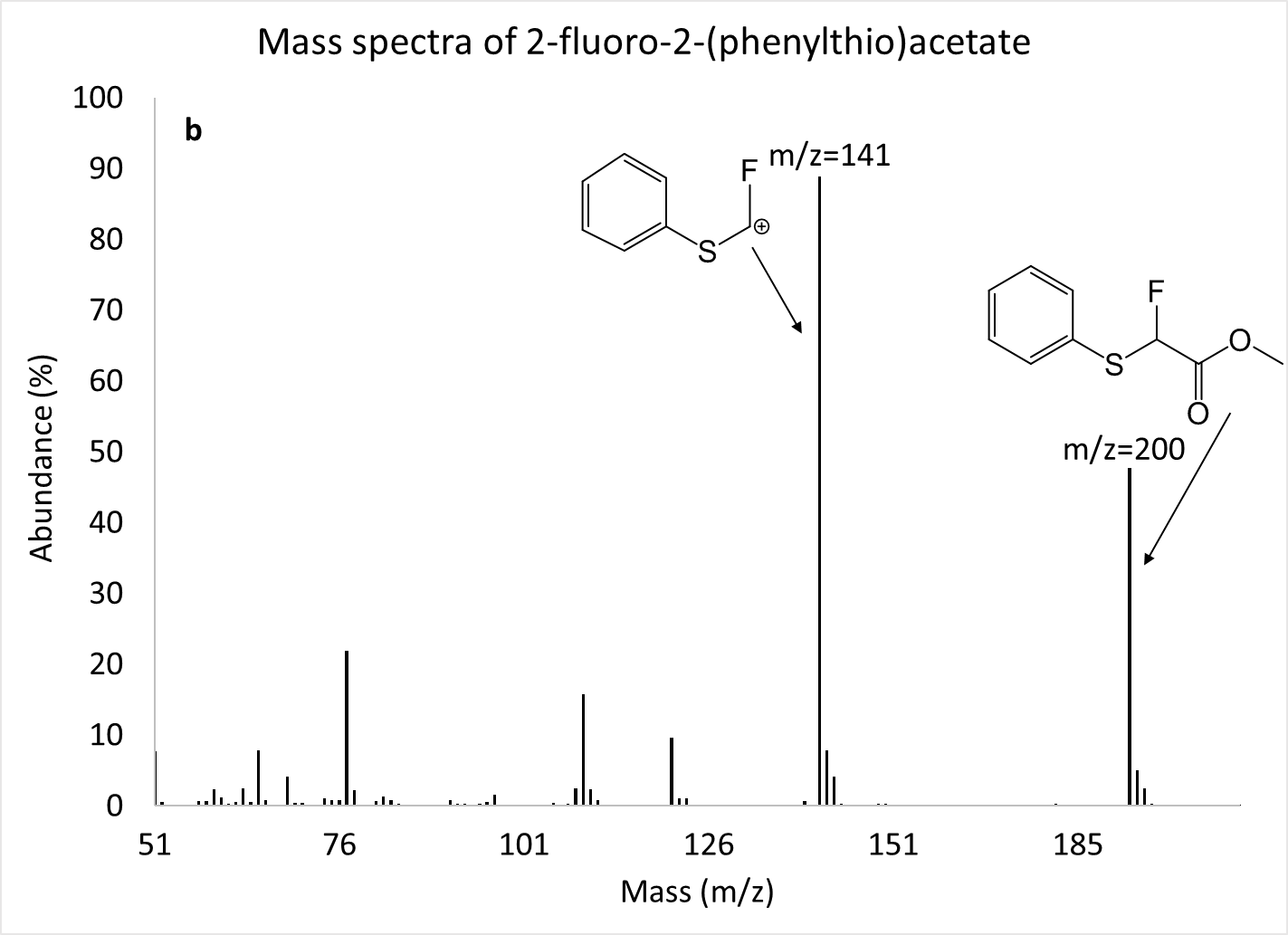 |

Figure 3. The a) GC-MS chromatogram and b) mass spectrum of the product **2**, purified post electrochemical synthesis using a semi-preparative HPLC column.


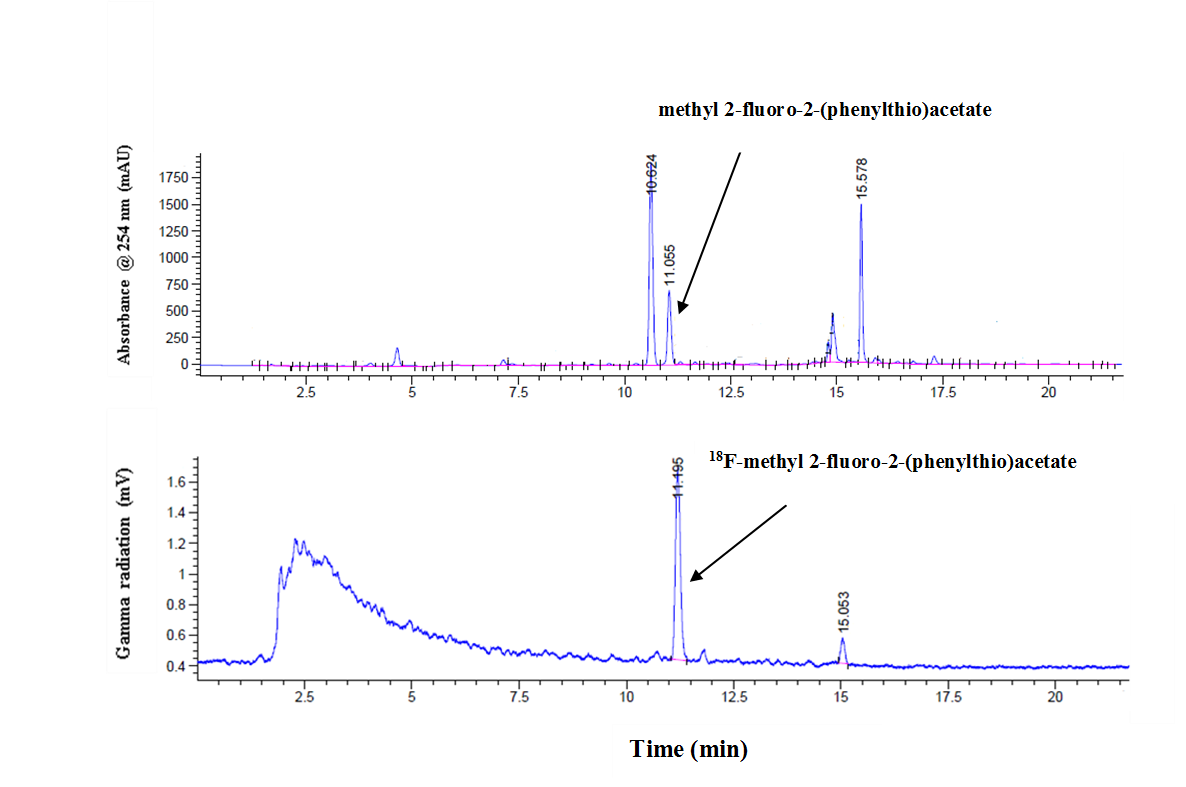
Figure 4. Analytical gamma + UV HPLC profiles of the crude sample after electrolysis. Electrolysis was performed for 30 min at 1.4 V vs Ag/Ag+ at 60 ºC using ACN solution containing 154 mM of TBAF, 25 mM of **1**, 104.6 mM of triflic acid and 5 mCi ^18^F-fluoride.


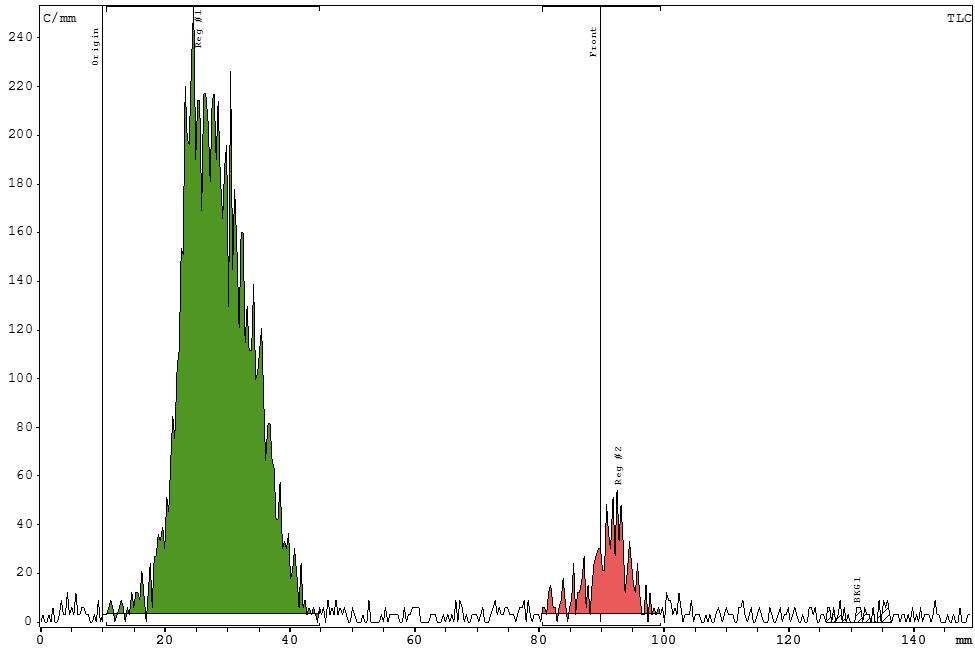


Nonreacted 18-F.

Radio-fluorinated organic compounds

Figure 5. Gamma TLC of the crude sample post radio-electrochemical synthesis. The electrolysis was performed for 30 min at 1.4 V vs Ag/Ag+ at 60 ºC using ACN solution containing 154 mM of TBAF, 25 mM of **1**, 104.6 mM of triflic acid and 5 mCi ^18^F-fluoride.

Figure 6. The proton NMR of the product **2** for further identification of the fluorinated product obtained by the electrochemical fluorination method.

Figure 7. Fluoride NMR of the same sample in figure 6.

**Reference**

1. Jouen, C., Lemaître, S., Lequeux, T. & Pommelet, J. C. Synthesis of α-fluoro-β-hydroxy alkylsulfanyl esters via a nucleophilic fluorination of sulfides. *Tetrahedron* **54,** 10801–10810 (1998).
